# Endogenous plasma activated protein C levels and the effect of enoxaparin and drotrecogin alfa (activated) on markers of coagulation activation and fibrinolysis in pulmonary embolism

**DOI:** 10.1186/cc9968

**Published:** 2011-01-17

**Authors:** Carl-Erik H Dempfle, Elif Elmas, Andreas Link, Nenad Suvajac, Volker Liebe, Jonathan Janes, Martin Borggrefe

**Affiliations:** 1 I. Department of Medicine, University Medical Center Mannheim, Theodor Kutzer Ufer, Mannheim, D-68167, Germany; 2 III. Department of Medicine, University Hospital of Homburg/Saar, Kirrberger Strasse, Homburg/Saar, D-66424, Germany; 3 Lilly Research Centre, London Road, Windlesham, GU20 6PH, UK

## Abstract

**Introduction:**

There are no published data on the status of endogenous activated protein C (APC) in pulmonary embolism (PE), and no data on the effect of drotrecogin alfa (activated) (DAA) given in addition to therapeutic dose enoxaparin.

**Methods:**

In this double-blind clinical trial, 47 patients with computed tomography (CT)-confirmed acute submassive PE treated with 1 mg/kg body weight of enoxaparin twice daily were randomized to groups receiving a 12-hour intravenous infusion of 6, 12, 18, or 24 μg/kg/hour of DAA or a placebo. Blood samples were drawn before starting DAA infusion, after 4, 8 and 12 hours (at the end of the infusion period), and on treatment days 2, 3, 4, 5 and 6.

**Results:**

Initial endogenous plasma activated protein C (APC) levels were 0.36 ± 0.48 ng/ml (<0.10 to 1.72 ng/ml) and remained in the same range in the placebo group. APC levels in patients treated with DAA were 13.67 ± 3.57 ng/ml, 32.71 ± 8.76 ng/ml, 36.13 ± 7.60 ng/ml, and 51.79 ± 15.84 ng/ml in patients treated with 6, 12, 18, and 24 μg/kg/hour DAA, respectively. In patients with a D-dimer level >4 mg/L indicating a high level of acute fibrin formation and dissolution, DAA infusion resulted in a more rapid drop in soluble fibrin, D-dimer, and fibrinogen/fibrin degradation products (FDP) levels, compared to enoxaparin alone. There was a parallel decline of soluble fibrin, D-dimer, FDP, and plasmin-plasmin inhibitor complex (PPIC) in response to treatment with enoxaparin ± DAA, with no evidence of a systemic profibrinolytic effect of the treatment.

**Conclusions:**

In patients with acute submassive PE endogenous APC levels are low. DAA infusion enhances the inhibition of fibrin formation.

**Trial registration:**

ClinicalTrials.gov: NCT00191724

## Introduction

Activated protein C inhibits blood coagulation by inactivating factors Va and VIIIa [1]. Inactivation of factor VIIIa reduces the activity of the tenase complex and the production of factor Xa. Inactivation of factor Va reduces the activity of the prothrombinase complex and the production of thrombin. Both mechanisms reduce the amount of thrombin and fibrin generated. *In vivo*, protein C is activated by the thrombin-thrombomodulin complex, which forms when thrombin binds to thrombomodulin on intact endothelium. Binding of thrombin to thrombomodulin also changes the specificity of thrombin from a procoagulant to an anticoagulant enzyme [2]. In addition to its effects on blood coagulation activation, activated protein C when bound to the endothelial protein C receptor (EPCR), activates protease-activated receptors (PARs), inducing a variety of cytoprotective cellular responses, including alteration of gene expression profiles, anti-inflammatory activities, anti-apoptotic activity, and endothelial barrier stabilization [3].

A high level of thrombin in a patient with a localized coagulation event such as venous thrombosis, and otherwise intact endothelium would be expected to result in elevated levels of activated protein C (APC), similar to what is observed in primates receiving an infusion of thrombin [4]. APC influences coagulation activation and organ dysfunction in animal models of sepsis [5]. No data have been published on the actual plasma levels of endogenous activated protein C in patients with acute PE.

Drotrecogin alfa (activated) (DAA) [6] is a recombinant form of human APC. Whereas endogenous production of activated protein C is dependent upon an ongoing coagulation process leading to the formation of thrombin, DAA levels achieved with infusion of DAA are independent of endogenous thrombin. Enoxaparin [7], a low molecular weight heparin commonly used for treatment of patients with acute deep vein thrombosis and pulmonary embolism, binds to antithrombin and changes its conformation to yield an effective inhibitor primarily of factor Xa. If enoxaparin and DAA are combined, this might result in a summation of anticoagulant effects. Alternatively, it is possible that the anticoagulant effect of therapeutic dose enoxaparin is maximal and cannot be enhanced by additional DAA therapy.

The reduction of thrombin-induced fibrin generation may lead to a drop in plasminogen activation by tPA, which is dependent upon the cofactor activity of fibrin [8,9] thus resulting in reduced fibrinolytic activity.

On the other hand, the anticoagulant effect of both drugs may result in an enhancement of fibrinolysis by reducing the amount of activated thrombin-activated fibrinolysis inhibitor (TAFIa) generated in the course of coagulation activation [10]. DAA may also have a profibrinolytic effect by binding PAI-1 and thus reducing PAI-1-capacity to inhibit tPA [11]. In fact, lower PAI-1 activity was detected in blood samples from patients treated with DAA compared to samples from patients treated with a placebo [12].

In the present study, we investigated the effect of therapeutic dose enoxaparin and four doses of DAA on blood coagulation status and markers of fibrin formation, activation of fibrinolysis, and fibrin dissolution in acute PE. This is the first clinical trial on the combination of a low molecular weight heparin at a therapeutic dose in combination with DAA, and the first study reporting endogenous APC levels in patients with acute PE.

## Materials and methods

### Inclusion and exclusion criteria

This was an exploratory, multicenter, randomized, parallel, double-blind, placebo-controlled phase II dose escalation study comparing a standard therapy for submassive pulmonary embolism (enoxaparin 1 mg/kg body weight twice daily by subcutaneous injection) to a combined therapy of DAA with enoxaparin. Patients were randomized according to a blinded randomization list held by the study coordinator. Patient identification numbers were obtained telephonically by the study physicians from the study coordinator. The trial was registered at ClinicalTrials.gov as NCT00191724. The study was started September 2004 and completed January 2008. The study was conducted in accordance with applicable laws and regulations, and ethical principles that have their origin in the Declaration of Helsinki. The institutional review boards of University Medical Center Mannheim and the other participating centers approved the study protocol, and all patients gave written informed consent.

The study was supported by Eli Lilly UK, Windlesham, Surrey, United Kingdom. This included funding for a study nurse; data management and statistics services provided by Koordinierungszentrum Klinische Studien (KKS) Heidelberg; trial medication and laboratory assays. Co-author Jonathan Janes is an employee of the Lilly Research Center, Windlesham, Surrey, United Kingdom.

Inclusion criteria were diagnosis of PE by spiral CT, clinical symptoms of acute PE for less than 48 hours, no massive PE judged as an indication for thrombolytic therapy, evidence of right ventricular dysfunction defined as right ventricular end-diastolic area/left ventricular end-diastolic area (RVEDA/LVEDA) ratio in the long axis greater than 0.6 associated with septal dyskinesia in the short axis [13], and age of ≥18 years. Exclusion criteria were: beginning of infusion of the study drug anticipated to be more than 24 hours after PE diagnosis by spiral CT, treatment with vitamin K antagonists in previous 5 days, pregnant or nursing women, major surgery within previous 24 hours, history of severe head trauma, intracranial surgery or stroke within the previous 3 months, evidence of intracerebral arteriovenous malformations or cerebral aneurysm, evidence of central nervous system mass lesion, neoplasm, or cerebral herniation, history of inherited or acquired chronic bleeding disorder, clinically significant gastrointestinal or genitourinary bleeding within the previous 6 weeks, clinical or laboratory evidence of hepatic failure, known esophageal varices, contraindications to enoxaparin for treatment of PE, history of heparin-induced thrombocytopenia type 2, femoral artery or subclavian artery puncture within the previous 48 hours, moribund patients expected to live not more than 24 hours, participation in another experimental interventional clinical trial within the previous 30 days, platelet count below lower limit of normal at inclusion, and creatinin clearance <30 ml/minute.

### Study treatment

After written informed consent and in addition to standard treatment with enoxaparin 1 mg/kg body weight twice daily, patients received a 12-hour continuous intravenous infusion of the study drug. A 12-hour infusion period was selected in order to limit the exposure to DAA because of safety concerns, since there was no prior experience with the combination of therapeutic dose enoxaparin or any other low molecular weight heparin, with DAA. Also, it was decided to start with a low dose of DAA and gradually increase the dose of DAA up to 24 μg/kg/hr, corresponding to the dose used in patients with severe sepsis.

Warfarin anticoagulation was initiated after Day 3. Enoxaparin treatment was terminated when therapeutic INR values of >2 were reached in response to warfarin.

Patients were randomly assigned to receiving DAA or a placebo as a study drug infusion. The study drug was prepared by a study pharmacist not involved in patient care and provided to the study physician in an infusion syringe labeled with the patient number and study identification. Group 1 included six patients treated with DAA at a dose of 6 μg/kg/hour and six patients receiving the placebo; group 2 included nine patients receiving DAA at a dose of 12 μg/kg/hour and three patients receiving the placebo; group 3 included nine patients treated with DAA at a dose of 18 μg/kg/hour and three patients receiving the placebo; and group 4 included eight patients treated with DAA at a dose of 24 μg/kg/hour and three patients receiving the placebo. Patients receiving the placebo from all phases of the study were combined for evaluation. After completion of each dose group, treatment and adverse event documentation were reviews and the safety evaluated by an independent data safety monitoring board (DSMB) before proceeding to the next dose of DAA. The study was terminated after inclusion of 47 of the originally planned 48 patients due to delays related to DSMB analysis and slow enrollment caused by competing trials.

The sample size was calculated to evaluate major bleeding. Assuming an approximate 5% rate with Enoxaparin there would be a >50% probability of detecting an additional event.

### Safety analyses

Safety analyses were based on the data from all 47 patients included. Hematology parameters (erythrocytes, hemoglobin level, leukocytes, platelets), prothrombin time (PT) and activated partial thromboplastin time (aPTT) were measured in fresh blood samples within four hours after blood sampling by the local laboratories of the participating centers. PT results were reported as Quick% and INR.

Safety endpoints included life-threatening bleeding, defined as fatal hemorrhage, reduction of hemoglobin level by >5 g/dl, hypotension caused by bleeding requiring inotropic support, intracranial hemorrhage, transfusion of >4 units of packed red blood cells, major bleeding defined as a decrease in hemoglobin levels of 2 to 5 g/dl, transfusion of two to four units of packed red blood cells, retroperitoneal bleeding, bleeding requiring surgical intervention, or development of hematomas requiring prolonged hospitalization, and minor bleeding defined as a decrease in hemoglobin of <2 g/dl, development of hematomas not requiring prolonged hospitalization, or blood transfusion of less than two units of packed red blood cells. Further safety endpoints were an aPTT more than three-fold the upper cutoff of normal range, recurrent pulmonary embolism or worsening of symptoms of pulmonary embolism requiring treatment with thrombolytic drugs, surgical or catheter embolectomy, occurrence of allergic reactions, diagnosis or heparin-induced thrombocytopenia type 2 (HIT-2), other types of thrombocytopenia, worsening of symptoms leading to endotracheal intubation and artificial ventilation, cardiopulmonary resuscitation, and death.

### Blood samples and laboratory analyses

Blood samples for preparation of citrated plasma were drawn immediately before starting the study drug infusion, 4, 8, and 12 hours after the start of the study drug infusion, and once daily on days 2, 3, 4, 5, and 6 of treatment. Special blood samples containing benzamidine for measurement of APC were drawn before the study drug infusion, and after 4, 8, and 12 hours.

A sufficient set of plasma and serum samples for the batch laboratory analyses was available from 12 patients treated with enoxaparin alone, all 6 patients of the DAA 6 μg/kg/hour group, 7 patients of the DAA 12 μg/kg/hour group, all 9 patients with the DAA 18 μg/kg/hour group, and 7 patients of the DAA 24 μg/kg/hour group, resulting in a total of 41 evaluable patients for the analysis of laboratory markers of coagulation and fibrinolysis activation. Samples were lost in two cases, and could not be used for laboratory analysis due to pre-analytical and handling mistakes in four cases.

The laboratory assays included prothrombin time (PT), aPTT, and anti-factor Xa chromogenic assay, using reagents and equipment from DadeBehring Diagnostics, Marburg, Germany. Fibrinogen was measured by turbidimetric immunoassay from Dako, Hamburg, Germany, using a Hitachi 904 autoanalyzer. Photometric immunoassays using antibody-coated latex particles were also performed on a Hitachi 904 autoanalyzer (Roche Diagnostics, Mannheim, Germany). The FDP-P assay for fibrinogen and fibrin degradation products was from Iatron Laboratories, Chiba, Japan. The Sekusui SF assay for measurement of soluble fibrin was from Daiichi Pure Chemicals, Ibaraki, Japan, and was also performed on the Hitachi 904 autoanalyzer. Plasmin-plasmin inhibitor complexes (PPIC, PAP) were measured using a 96-well microtiter plate ELISA from DRG Instruments GmbH, Marburg, Germany.

APC was measured using the enzyme capture assay of Gruber and Griffin [14] with minor modifications. The lower limit of detection of the assay was 0.5 ng/mL.

### Statistical analyses

Statistical analyses involved calculation of means, standard deviations, medians, interquartile ranges. In order to minimize the effect of outliers and distribution effects in view of the small number of patients, medians were used rather than mean values for the line graphs. All group comparisons were performed using Wilcoxon's signed rank sum test. For correlation graphs, coefficients of correlation R were calculated, using a linear regression model.

## Results

Table 1 contains the baseline characteristics of patients enrolled in the study by treatment group. There were imbalances in baseline characteristics that are likely due to the small number of patients enrolled in each treatment group. Patients who were enrolled earlier and received the lower dosages of DAA tended to be older than patients enrolled later in the study. Right ventricular dysfunction was present at admission in all patients, as this was an entry criterion. Right ventricular end-diastolic area divided by left ventricular end-diastolic area (RVEDA/LVEDA) was used as an indicator of right ventricular dysfunction. A value of >0.6 was considered to be pathologic. Mean and median values of RVEDA/LVEDA ratio decreased during treatment in all groups, with no obvious differences between patients receiving DAA or placebo (Table 2). RVEDA/LVEDA ratios were calculated on the basis of echocardiography examinations performed at admission, after 6 days, and after 90 days.

**Table 1:** Baseline characteristics of patients included (means ± standard deviation, range)

Variable	DAA 6 μg/kg/h	DAA 12 μg/kg/h	DAA 18 μg/kg/h	DAA 24 μg/kg/h	Placebo
n	6	9	9	8	15
Age (years)	70.8 ± 4.4 (66.0 to 78.0)	65.7 ± 8.9 (46.0 to 74.0)	51.9 ± 16.5 (30.0 to 72.0)	45.6 ± 22.6 (18.0 to 78.0)	60.7 ± 21.9 (22.0 to 84.0)
Sex (Female)	3/6	3/9	5/9	3/8	11/15
Body weight (kg)	76.0 ± 9.7 (60.0 to 85.0)	93.9 ± 13.3 (79.0 to 120.0)	85.2 ± 17.6 (66.0 to 114.0)	86.4 ± 13.8 (62.0 to 104.0)	85.7 ± 18.6 (50.0 to 113.0)
Systolic blood pressure (mmHg)	115.0 ± 15.8 (100.0 to 140.0)	129.1 ± 20.4 (95.0 to 160.0)	129.1 ± 30.7 (95.0 to 188.0)	119.5 ± 16.7 (80.0 to 132.0)	133.7 ± 25.4 (100.0 to 181.0)
Diastolic blood pressure (mmHg)	66.4 ± 10.4 (57.0 to 80.0)	86.4 ± 17.5 (62.0 to 120.0)	82.4 ± 12.7 (70.0 to 104.0)	68.9 ± 12.0 (50.0 to 85.0)	76.6 ± 14.3 (60.0 to 110.0)
Highest heart rate (1/minute)	83.5 ± 14.2 (60.0 to 100.0)	95.2 ± 19.4 (80.0 to 140.0)	94.4 ± 17.3 (75.0 to 127.0)	105.4 ± 21.6 (70.0 to 130.0)	106.6 ± 9.9 (95.0 to 130.0)
Earlier PE	1/6	2/9	0	1/8	0
Earlier DVT	1/6	2/9	1/9	2/8	2/15
Earlier ischemic stroke	1/6	1/9	0	0	3/15

DAA, Drotrecogin alfa (activated); DVT, deep vein thrombosis; PE, pulmonary embolism.

**Table 2:** RVEDA/LVEDA ratio

Variable	DAA 6 μg/kg/h	DAA 12 μg/kg/h	DAA 18 μg/kg/h	DAA 24 μg/kg/h	Placebo
RVEDA/LVEDA Day 0	0.8 ± 0.3 (0.6 to 1.4)	1.0 0.3 (0.7 to 1.6)	1.0 0.4 (0.7 to 1.7)	0.9 ± 0.4 (0.6 to 1.6)	1.1 0.5 (0.6 to 2.8)
	*N *= 6	*N *= 9	*N *= 9	*N *= 8	*N *= 15
RVEDA/LVEDA Day 6	0.7 ± 0.3 (0.5 to 1.2)	0.7 ± 0.2 (0.5 to 1.0)	0.8 ± 0.3 (0.5 to 1.5)	0.6 ± 0.2 (0.4 to 1.0)	0.7 ± 0.2 (0.5 to 1.1)
	*N *= 5	*N *= 8	*N *= 8	*N *= 7	*N *= 15
RVEDA/LVEDA Day 90	0.5 ± 0.1 (0.4 to 0.7)	0.5 ± 0.1 (0.4 to 0.6)	0.6 ± 0.1 (0.4 to 0.6)	0.5 ± 0.1 (0.4 to 0.6)	0.6 ± 0.2 (0.3 to 0.9)
	*N *= 5	*N *= 8	*N *= 8	*N *= 5	*N *= 11

DAA, Drotrecogin alfa (activated); LVEDA, levt ventricular enddiastolic area; RVEDA, right ventricular enddiastolic area.

All patients were treated with therapeutic dose enoxaparin, which led to elevated anti-factor Xa activity levels within the therapeutic range for enoxaparin, with no significant differences between DAA treatment groups. Mean value was 0.66 ± 0.16 aXa U/mL during DAA treatment phase (range 0.38 to 1.06 aXa U/mL).

Laboratory results of the samples drawn before the start of the study drug infusion are shown in Table 3. All patients displayed abnormal D-dimer levels, as well as elevated levels of soluble fibrin, fibrinogen/fibrin degradation products and PPIC.

**Table 3:** Laboratory values before start of study medication

Parameter	Mean	SD	Median	Min	Max
APC (ng/mL)	0.36	0.48	0.00	0.00	1.72
PT Quick (%)	92	11	93	75	119
INR	1.05	0.08	1.00	0.90	1.20
aPTT (sec)	30	13	26	22	89
Fibrinogen (g/L)	3.10	1.01	3.08	1.54	6.61
TINAquant D-dimer (mg/L)	7.19	4.25	6.80	0.76	15.53
Sekisui SF (mg/L)	33.73	20.84	33.75	11.10	125.10
Iatron FDP-P (mg/L)	23.02	20.78	18.80	4.30	105.10
PPIC (μg/L)	1,022	731	777	219	3,217

APC, activated protein C; aPTT, activated partial thromboplastin time; FDP-P, fibrinogen/fibrin degradation products in plasma; INR, international normalized ratio; PPIC, plasmin plasmin inhibitor complex; PT, prothrombin time; SF, soluble fibrin.

**Figure 1:**
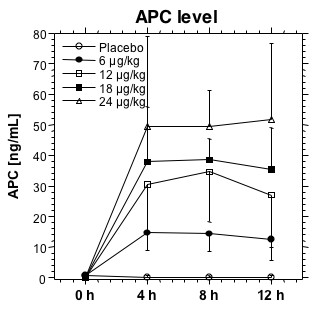
**APC activity at 0, 4, 8 and 12 hours (end of study drug infusion)**. Course of APC activity at inclusion, after 4 hours, 8 hours and after 12 hours (end of the study drug infusion). Patients receiving placebo as the study drug infusion displayed low APC activity levels. DAA infusion results in a dose-dependent increase in APC activity levels.

Despite the high level of coagulation activation present in patients with acute pulmonary embolism, levels of endogenous APC were low (Table 3, and Figure 1). Values were below the detection limit of 0.5 ng/mL in the majority of patients. In the patients treated with enoxaparin alone, values did not change. Infusion of DAA led to a dose-dependent increase in APC levels (Figure 1). The APC levels attained were above the physiological range in all dose groups and remained constant during the infusion period of 12 hours. APC levels in patients treated with DAA were 13.67 ± 3.57 ng/ml, 32.71 ± 8.76 ng/ml, 36.13 ± 7.60 ng/ml, and 51.79 ± 15.84 ng/ml in patients treated with 6, 12, 18, and 24 μg/kg/hour DAA, respectively.

Three patients of the 6 μg/kg group and one patient of the 12 μg/kg group had been treated with a bolus dose of unfractionated heparin (UFH) initially, which caused prolongation of aPTT in the pre-DAA-treatment plasma sample. For analysis of the effect of DAA on aPTT, but not for all other analyses; these patients were excluded.

**Figure 2:**
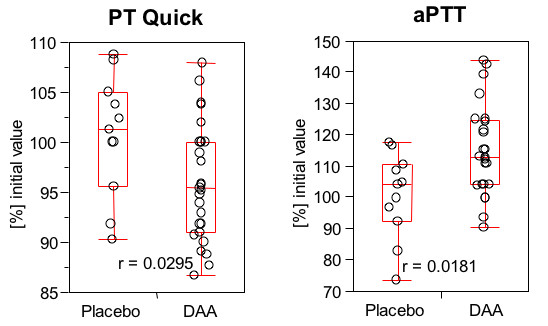
**Prothrombin time (PT Quick percent) and aPTT 12 hours after start of infusion**. Prothrombin time (PT Quick percent) and aPTT 12 hours after the start of the study drug infusion. DAA infusion caused a prolongation of PT (reduction in Quick percent ratio) and aPTT.

Infusion of DAA caused a transient increase in prothrombin time (resulting in a reduced Quick percent ratio) and aPTT. Figure 2 shows the results of the 12-hour sample drawn at the end of DAA infusion. Median maximal aPTT levels were approximately 115% of the initial value at the highest DAA dose. After termination of DAA infusion, PT and aPTT returned to pre-DAA treatment levels. These results indicate a detectable additional anticoagulant effect induced by DAA given in addition to therapeutic dose enoxaparin in patients with acute PE. Since conventional citrated plasma was used for these analyses, the actual *in vivo *effect is expected to be greater, due to the short *in vitro *half-life of DAA.

**Figure 3:**
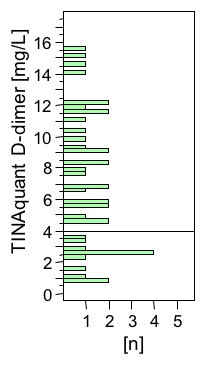
**TINAquant D-dimer levels in sample drawn immediately before study drug infusion**. Distribution of TINAquant D-dimer levels (0 h sample drawn immediately before the start of the study drug infusion).

The distribution of D-dimer levels of all patients is shown in Figure 3. Three of 12 patients in the placebo group, 2 of 6 patients in the 6 μg/kg BW group, 2 of 7 patients in the 12 μg/kg BW group, 2 of 9 patients in the 18 μg/kg BW group, and 3 of 7 patients in the 24 μg/kg BW group displayed TINAquant D-dimer values of <4 mg/L in the baseline plasma samples. For analysis of the effect of DAA on fibrin formation and fibrinolysis, these patients were excluded, because calculation of a relative decrease (percent of initial value) led to a disproportional effect of low initial values on the final results. For the analysis, patients treated with DAA were combined in one group. This resulted in a population of 9 patients in the placebo group and 20 patients treated with DAA.

**Figure 4:**
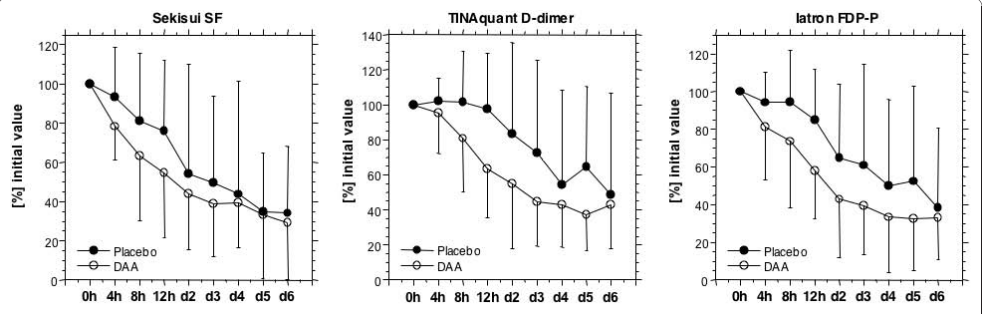
**Course of Sekisui soluble fibrin, Tinaquant D-dimer, and Iatron FDP-P**. Sekisui soluble fibrin, Tinaquant D-dimer, and Iatron FDP-P before the study drug infusion, after 4, 8 and 12 hours, and on days 2, 3, 4, 5 and 6 days for all patients with an initial (0 h) TINAquant D-dimer level of >4 mg/L. Individual initial values were set at 100% to compensate for individual differences in levels. Initiation of anticoagulant therapy results in a drop in all fibrin-related markers, DAA infusion accelerates the decline of the fibrin-related markers.

Treatment of patients with acute submassive PE with enoxaparin caused a rapid decrease in markers of fibrin formation and fibrin dissolution (Figure 4). There is no obvious profibrinolytic effect, as soluble fibrin, D-dimer, and fibrinogen/fibrin degradation products decrease in parallel.

**Figure 5:**
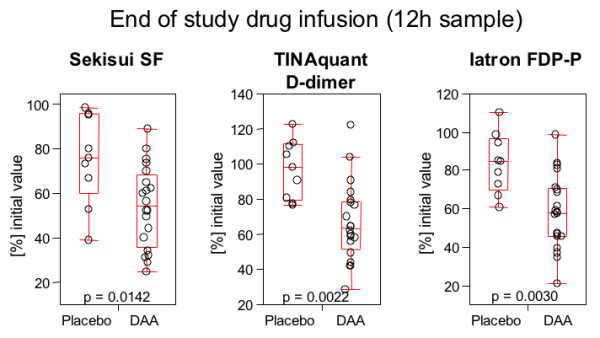
**Sekisui soluble fibrin, TINAquant D-dimer, and Iatron FDP-P at end of study drug infusion**. Sekisui soluble fibrin, Tinaquant D-dimer, and Iatron FDP-P: comparison of the results of the 12-hour sample for all patients with an initial TINAquant D-dimer level of >4 mg/L Patients receiving DAA displayed significantly lower levels of fibrin-related markers at the end of the study drug infusion.

Addition of DAA to enoxaparin in the initial treatment phase resulted in a more rapid decline in soluble fibrin, D-dimer, and fibrinogen/fibrin degradation products, compared to enoxaparin alone, in patients with an initial D-dimer level of >4.0 mg/L (Figure 4). As shown in Figure 5, the difference is statistically significant for the 12-h sample drawn at the end of the DAA infusion period.

**Figure 6:**
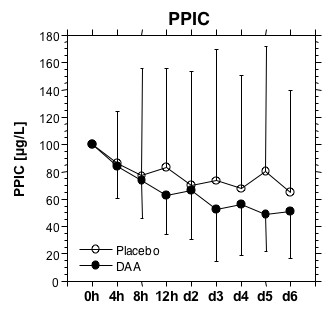
**Plasmin-plasmin inhibitor complex (PPIC)**. Plasmin-plasmin inhibitor complex (PPIC) levels before the study drug infusion, after 4, 8 and 12 hours, and on days 2, 3, 4, 5 and 6 days. Individual initial values were set at 100% to compensate for individual differences in levels. Initiation of anticoagulant therapy results in a reduction in PPIC values, indicating a lower plasmin generation compared to initial levels.

Plasmin-plasmin inhibitor complexes (PPIC) decline in parallel to soluble fibrin, and the fibrin degradation products, with no obvious effect of DAA (Figure 6).

There were no significant changes in hemoglobin, hematocrit, or leukocyte count during enoxaparin therapy. DAA treatment also had no effect on these parameters.

Bleeding complications were within the expected range for full-dose enoxaparin therapy. No patient experienced life-threatening bleeding. Two patients experienced major bleeding after infusion of DAA: One patient in the 6 μg/kg/hour DAA group suffered from intracranial hemorrhage on Day 4 of treatment, associated with a drop in hemoglobin level >2 g/L. One patient in the placebo group showed a drop in hemoglobin level by >5 g/L. DAA treatment did not appear to increase the risk of bleeding in any of the dose groups studied.

## Discussion

In healthy persons, APC levels are in the range of 1 to 3 ng/ml [14]. Patients with systemic coagulation activation but normal endothelial function display APC levels as high as 50 to 80 ng/ml. In patients with severe sepsis, which also have strongly elevated levels of D-dimer and other fibrin-related markers, APC levels are generally in the range of 10 to 20 ng/ml [15]. In the patients with acute submassive PE, the intravascular fibrin formation is not associated with elevated levels of endogenous APC. Baseline endogenous APC levels are low, and remain in the low range during treatment with enoxaparin.

In patents with severe sepsis, treatment with activated protein C may improve clinical outcome, by reducing organ dysfunction due to microvascular occlusion and other mechanisms [16,17]. In view of the impaired protein C system present in many patients with PE, we hypothesized that treatment with DAA might lead to more effective anticoagulation and improved activation of fibrinolysis, compared to therapy with enoxaparin alone.

In the present investigation, DAA treatment leading to supraphysiological levels of APC had an additional anticoagulant effect, associated with a prolongation of prothrombin time and aPTT during the 12 hours of infusion. Given the short plasma half-life of APC of approximately 25 minutes, the ability to show an anticoagulant effect will be dependent on the speed of sample preparation and analysis and the actual *in vivo *effect might be greater. Petäjä *et al. *described a synergistic effect of unfractionated heparin and APC concerning aPTT [18]. As therapeutic range enoxaparin has only minimal effect on prothrombin time and aPTT; the effect found in the present study can be attributed to DAA alone.

The *in vivo *effects of anticoagulants on coagulation are reflected by markers of fibrin formation and fibrin dissolution. The Sekisui SF assay specifically detects non-plasmin degraded fibrin monomer complexes [19]. TINAquant D-dimer is specific for plasmin-degraded crosslinked fibrin [20]. In addition, we used a quantitative fibrinogen/fibrin degradation product assay for analysis of the status of intravascular fibrin formation and fibrin dissolution.

Anticoagulant therapy with enoxaparin blunted intravascular fibrin formation, leading to a decline in soluble fibrin levels. D-dimer and fibrinogen/fibrin degradation product levels declined in parallel, indicating a close association between intravascular fibrin formation and fibrin dissolution in the patients with submassive PE. DAA enhances the anticoagulant effect of enoxaparin, leading to more rapid decline in soluble fibrin and the other fibrin-related markers in patients with high levels of these fibrin-related markers.

The currently approved therapeutic dose of 24 μg/kg/hr (for 96 hours) of DAA for the treatment of severe sepsis patients partially (reduced by about 25%) blunted thrombin formation, as evidenced by reduction of D-dimer levels, prothrombin fragment F1.2, and thrombin-antithrombin complex [12,16].

In a human model of low dose endotoxemia, DAA alone at a dose of 24 μg/kg/hr was unable to reduce coagulation activation [21,22]. In this same model of human low dose endotoxemia, low molecular weight heparin and unfractionated heparin almost totally suppressed coagulation activation [23,24]. The combination of DAA and therapeutic dose enoxaparin has not been investigated in this model.

There were no signs of a systemic profibrinolytic effect of enoxaparin, or the combination of enoxaparin with DAA. The lack of a profibrinolytic effect of enoxaparin or DAA combined with enoxaparin was also obvious in the results of the PPIC assay. Levels of PPIC dropped in parallel to the soluble fibrin levels, emphasizing the role of soluble fibrin as cofactor in plasminogen activation [25,26].

Apart from the safety aspect, the aim of the study was to detect short-term effects of DAA on markers of fibrin formation and fibrin dissolution in patients with acute submassive PE. The study was not intended to show clinical efficacy, and clinical evaluation was focused primarily on safety issues such as occurrence of bleeding. The incidence of major bleeding was low and within the expected range for therapeutic dose enoxaparin alone. One of the two cases of severe bleeding occurred in the group receiving no DAA.

## Conclusions

Coagulation activation in acute submassive PE does not lead to a systemic activation of protein C. Treatment with enoxaparin causes a parallel reduction in soluble fibrin and fibrin degradation products, with no obvious profibrinolytic effect. Addition of DAA causes a more rapid decline in fibrin-related markers, but does not change PPIC levels, or the relationship between markers of fibrin formation and fibrin dissolution. A profibrinolytic effect of anticoagulants in PE leading to more rapid clot dissolution appears to be a local effect at the site of the embolus rather than a systemic phenomenon. Further studies are needed to investigate a potential clinical benefit related to application of DAA in acute thromboembolic events. A longer time frame of DAA application might result in more pronounced effects.

## Key messages

• Patients with an acute submassive pulmonary embolism do not display elevated levels of endogenous activated protein C, despite a high level of coagulation activation and presence of intact endothelium.

• Treatment with therapeutic dose enoxaparin reduces the level of coagulation activation, with a drop in the levels of soluble fibrin complexes, D-dimer antigen, and fibrinogen/fibrin degradation products.

• Recombinant human activated protein C (Drotrecogin alfa (activated)) accelerates suppression of coagulation activation in patients with high levels of intravascular fibrin.

• Neither enoxaparin, nor the combination of enoxaparin with Drotrecogin alfa (activated) induces a systemic profibrinolytic response.

## Abbreviations

APC: Activated protein C; aPTT: Activated partial thromboplastin time; BW: Body weight; CT: Computerized tomography; DAA: Drotrecogin alfa (activated); recombinant activated protein C; DSMB: Data safety monitoring board; EPCR: Endothelial protein C receptor; FDP: Fibrinogen/fibrin degradation products; HIT-2: Heparin-induced thrombocytopenia type 2; KKS: Koordinierungszentrum klinische Studien (coordinating center for clinical trials); LVEDA: Left ventricular enddiastolic area; PAI-1: Plasminogen activator inhibitor-1; PAP: Plasmin-Antiplasmin complex; PAR: Protease-activated receptor; PE: Pulmonary embolism; PPIC: Plasmin-plasmin inhibitor-complex; PT: Prothrombin time; RVEDA: Right ventricular enddiastolic area; SF: Soluble fibrin; tPA: Tissue plasminogen activator.

## Competing interests

Co-author Jonathan Janes is an employee of the Lilly Research Center, Windlesham, Surrey, United Kingdom. All other authors declare that they have no competing interests.

## Authors' contributions

CED developed the trial design, recruited and treated patients, supervised the laboratory analyses and statistical evaluation, and wrote the manuscript. EE, AL, NS, and VL recruited and treated study patients. JJ was involved in data evaluation and interpretation, and writing of the manuscript. MB supervised trial performance. All authors read and approved the final manuscript.
